# Supplementary material for: A set of multi-entry identification keys to African frugivorous flies (Diptera, Tephritidae)
Source: Zookeys. 2014 Jul 24;(428):97–108. doi: 10.3897/zookeys.428.7366 (PMC4143993; doi:10.3897/zookeys.428.7366)
Supplement: Supplementary material 6 — Key to Ceratitis [file zookeys-428-097-s006.zip › SF6_ZooKeys_key to Ceratitis/key/SF6_ZooKeys_key to Ceratitis/Media/Html/Ceratitis sucini .htm]

Ceratitis (Ceratalaspis) sucini sp


***Ceratitis*** **(*Ceratalaspis*)
*sucini* De Meyer**

*Ceratitis* (*Ceratalaspis*)
*sucini* De Meyer, 1998 : 287.

Body
length: 5.85 mm; wing length: 5.40 mm.

Female

Head.
Antenna yellow-orange. Third antennal segment two times as long as second
segment. Arista with short hairs over entire length. Frons yellow; with short
scattered hairs which are largely the same colour as the frons; in lateral view
not distinctly projecting forwards at antennal implant. Chaetotaxy normal for
subgenus.

Thorax.
Postpronotum white; unspotted. Ground colour of mesonotum pale. Chaetotaxy
normal for subgenus. Scapular setae pale. One anepisternal bristle. Scutellum
yellow-white; with three black separate markings; basally without distinct
spots. Subscutellum pale with three dark separate spots.

Legs
yellow; setation typical for subgenus, mainly pale especially on femora.
Posterior and posterodorsal rows on fore femur pale. Ventral spines on fore
femur reddish.

Wing
bands with markings extensively yellow. Banding, marginal band continuous;
cubital band free; medial band joined with marginal band; crossvein r-m just
before middle of discal cell. Crossvein dm-cu posteriorly more inwards than
anteriorly.

Abdomen.
Dark yellow. Setation and banding typical for subgenus. Oviscape shorter than
abdomen.

Male

Unknown.�

(Description
after De Meyer, 1998)
